# Effectiveness of physical exercise on foot pain and function in adults with rheumatoid arthritis: systematic review and meta-analysis

**DOI:** 10.1007/s10067-026-08044-8

**Published:** 2026-03-21

**Authors:** Alejandro Cruz-López, Ana María Rayo-Pérez, Natalia Tovaruela-Carrión, Priscila Távara-Vidalón, Pedro V. Munuera-Martínez

**Affiliations:** Department of Podiatry, Faculty of Nursing, Physiotherapy and Podiatry, University of Seville, C/Avicena s/n., 41009 Seville, Spain

**Keywords:** Foot pain, Inflammation, Meta-analysis, Physical function, Rehabilitation, Rheumatoid arthritis, Therapeutic exercise

## Abstract

**Background:**

Foot involvement is highly prevalent in rheumatoid arthritis (RA), affecting over 90% of patients during the disease course. However, the specific impact of structured exercise on foot pain and functional limitations remains insufficiently understood. This systematic review and meta-analysis aimed to evaluate the effectiveness of supervised exercise programs on foot-specific outcomes in adults with RA.

**Methods:**

We conducted a systematic review and meta-analysis following PRISMA guidelines, searching five databases for randomized and controlled quasi-experimental trials evaluating supervised exercise interventions in adults with RA and foot involvement. Primary outcomes included foot pain and physical function measures. Data were pooled using random-effects models, and risk of bias was assessed using Cochrane tools. Analysis was performed with RevMan 5.4 and STATA 17.

**Results:**

Thirteen studies (*n* = 548) were included; ten entered the meta-analysis. Exercise significantly reduced foot pain (SMD − 0.68, 95% CI − 0.89 to − 0.46; *p* < 0.001) and improved function (Health Assessment Questionnaire SMD − 0.73, 95% CI − 0.96 to − 0.49; 6MWT MD + 47.6 m, 95% CI 31.4 to 63.8; Time Up-and-Go SMD − 0.40, 95% CI − 0.59 to − 0.21). Aquatic exercise and Tai Chi showed larger pain reductions, while high-intensity interval training improved functional outcomes. Programs ≥ 12 weeks yielded greater effects. Risk of bias ranged from low to some concerns; non-randomized studies showed moderate–serious confounding risk.

**Conclusions:**

Supervised, structured exercise reduces foot pain and improves function in RA, with aquatic and combined modalities particularly beneficial. Findings support implementation within multidisciplinary care.

## Introduction

Rheumatoid arthritis (RA) is a long-term autoimmune condition marked by ongoing inflammation of the synovial joints, causing pain, stiffness, and gradual joint deterioration that can result in functional impairment. Its etiology is multifactorial, involving genetic predisposition, environmental factors such as smoking, and possible alterations in the gut microbiome that may trigger abnormal immune responses [1–3]. Globally, RA affects 0.24% to 0.50% of the population, with higher prevalence among women and in high-income countries [4, 5].

Foot involvement is highly prevalent, occurring in more than 90% of patients during the disease course. The metatarsophalangeal, subtalar, and talonavicular joints are commonly affected, often from early stages, leading to deformities such as hallux valgus, claw toes, medial arch collapse, and ankle stiffness [6, 7]. These changes compromise gait, balance, and independence. Despite their clinical significance, foot and ankle joints are frequently excluded from widely used indices such as the Disease Activity Score in 28 joints (DAS28), which may underestimate their contribution to disease burden and limit tailored interventions [8].

Pharmacological treatment with disease-modifying antirheumatic drugs (DMARDs), both conventional and biologic, remains the cornerstone of RA management. Yet, many patients continue to experience pain, stiffness, and impaired function, particularly in the feet [9, 10]. This highlights the importance of non-pharmacological strategies, especially therapeutic exercise, within multidisciplinary care.

Evidence indicates that exercise interventions, including aerobic training, resistance exercise, balance training, stretching, and mind–body practices such as yoga or Pilates, can improve function, reduce pain, and enhance quality of life without exacerbating inflammation [11–13]. However, most studies evaluate outcomes in a generalized manner, with limited focus on foot-specific pain and function, despite the central role of the feet in mobility and daily activity.

Given the high prevalence and clinical impact of foot involvement in RA, a systematic review is warranted to clarify the effects of physical exercise on foot pain and function. The primary objective of this review and meta-analysis is to synthesize available evidence on this relationship. Secondary objectives are to compare the effects of different exercise modalities on foot health and to evaluate the influence of program intensity and duration to identify optimal prescription parameters.

## Methods

### Study design

This study was conducted as a systematic review and meta-analysis in accordance with the PRISMA 2020 (Preferred Reporting Items for Systematic Reviews and Meta-Analyses) guidelines [14]. The protocol was prospectively registered in the PROSPERO database (CRD420250632483) to ensure methodological transparency and minimize reporting bias. For this study, ethical committee approval was not required because it is a systematic review and meta-analysis of previously published data.

### PICO question

In this study, the PICO question was developed as follows:

- **Participants (P):** Adults (≥ 18 years) with a confirmed diagnosis of RA according to the American College of Rheumatology (ACR) and/or the European Alliance of Associations for Rheumatology (EULAR) classification criteria and reported foot involvement, defined as foot pain, deformity, and/or functional limitation. Concomitant use of immunomodulatory or biologic therapies was permitted.
- **Interventions (I):** Interventions comprised structured, supervised exercise programs of at least four weeks’ duration, with clearly specified type, frequency, intensity, and progression. Eligible modalities included aquatic exercise, progressive resistance training, adapted high-intensity interval training (HIIT), and Tai Chi.
- **Comparison (C):** Usual care, attention control, waitlist, or alternative non-exercise interventions.
- **Outcomes (O):** Studies had to report at least one quantitative outcome in the following domains: pain (Visual Analogue Scale (VAS) or Western Ontario and McMaster Universities Osteoarthritis Index (WOMAC) pain subscale), function (Health Assessment Questionnaire (HAQ), 6-min walk test (6MWT), or Timed Up-and-Go (TUG).

### Eligibility criteria

We included randomized controlled trials (RCTs) and prospective controlled quasi-experimental studies that evaluated supervised, structured exercise programs (≥ 4 weeks) in adults diagnosed with RA. The eligible comparators were usual care, attention control, waitlist, or alternative non-exercise interventions. Studies were required to report at least one quantitative primary outcome related to foot pain (VAS or WOMAC) or foot function (HAQ, 6MWT or TUG). Secondary outcomes included foot-related biomechanical, inflammatory, or quality-of-life measures.

Foot involvement was defined pragmatically based on the eligibility criteria of the included studies rather than on a single standardized definition. Studies were considered eligible if participants reported foot or ankle pain, foot-related symptoms, or functional limitations in which foot involvement was likely to play a relevant role. As a result, foot involvement was primarily inferred from self-reported pain measures targeting the foot region and from functional outcomes known to be influenced by foot pain, deformity, or instability.

We excluded single-group pre-post studies, case series, conference abstracts, meta-analyses, and studies where exercise was embedded within a broader multi-component intervention, preventing the isolation of its specific effect. Studies not published in English were also excluded. Studies that did not provide sufficient statistical data (means, standard deviations, sample sizes) to permit the calculation of effect sizes for meta-analysis were also excluded. Studies that did not meet these quantitative eligibility criteria were excluded from the meta-analysis but considered in the narrative synthesis when relevant.

### Search strategy and study selection

Searches were conducted in MEDLINE/PubMed, Scopus, Cochrane Library, Web of Science, and CINAHL for randomized controlled trials and controlled quasi-experimental studies evaluating the effectiveness of exercise on foot pain and function in patients with rheumatoid arthritis, covering the period from January 1980 through July 31, 2025. The start date was chosen to reflect contemporary exercise prescription practices and minimize historical heterogeneity. The search strategy combined controlled vocabulary (MeSH terms) and free-text terms. A representative PubMed search strategy was: (“Arthritis, Rheumatoid”[Mesh] OR “rheumatoid arthritis”[tiab]) AND (“Exercise”[Mesh] OR “Exercise Therapy”[Mesh] OR “Tai Ji”[Mesh]) AND (“Foot”[Mesh] OR “foot”[tiab] OR “podal”[tiab]) AND (“Pain”[Mesh] OR “pain”[tiab] OR “functionality”[tiab] OR “HAQ”[tiab]). Additionally, reference lists of eligible studies and relevant systematic reviews were hand-searched to identify additional records.

**Fig. 1:**
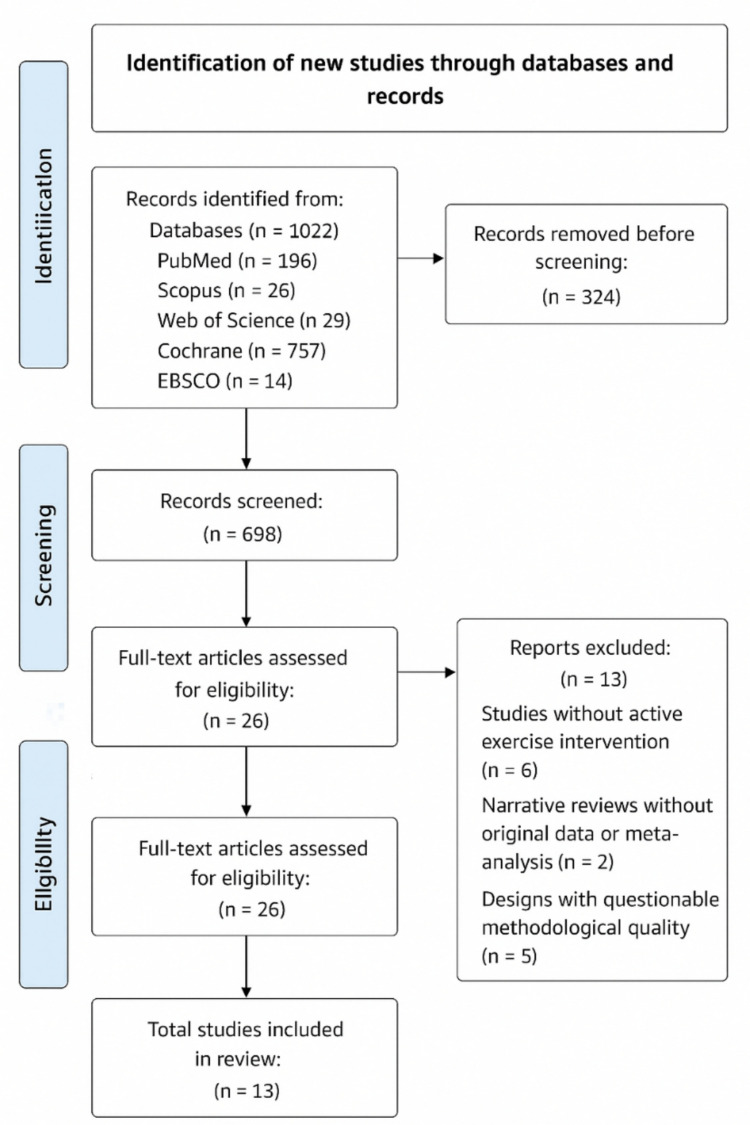
PRISMA flow diagram

The study selection process was performed in three phases: (1) removal of duplicates using Zotero, (2) independent screening of titles and abstracts by two reviewers using Rayyan QCRI, and (3) full-text review of potentially eligible articles to confirm methodological adequacy, target population, intervention type, and outcome measures. Discrepancies were resolved by consensus or, when necessary, adjudication by a third reviewer. The selection process is summarized in a PRISMA flow diagram (Fig. 1).

### Data extraction

Two reviewers independently extracted data using an Excel form. The extracted data included basic information (authors, publication year, country, study design, sample size), intervention characteristics (exercise modality, frequency, intensity, duration), clinical outcomes (foot pain, functional measures, and other relevant biomarkers), and risk-of-bias assessment. Any discrepancies in extracted data were resolved by consensus between the two reviewers, with involvement of a third reviewer when necessary.

### Risk of bias assessment

**Fig. 2:**
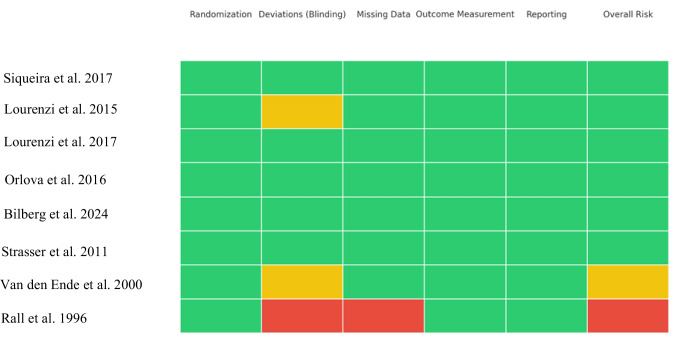
Risk of bias summary for the included randomized controlled trials, assessed using the Cochrane RoB 2 tool. Legend: colors reflect domain-level risk of bias (RoB 2). Green = low risk; yellow = some concerns; red = high risk

**Fig. 3:**
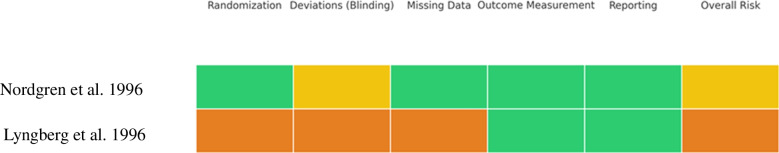
Risk of bias assessment for the included non-randomized controlled studies, evaluated using the ROBINS-I tool. Legend: colors reflect domain-level risk of bias (RoB 2). Green = low risk; yellow = some concerns; orange = serious; red = high risk

The risk of bias was independently evaluated by two reviewers according to the type of study design. For randomized controlled trials, the Cochrane RoB 2 tool was utilized, assessing five key domains: the randomization process, deviations from intended interventions, missing outcome data, outcome measurement, and selection of reported results (Fig. 2). For non-randomized controlled studies, the Risk Of Bias In Non-randomized Studies of Interventions (ROBINS-I) tool was applied (Fig. 3).

### Statistical analysis

Outcomes related to foot pain and function were combined using standardized mean differences (SMD) or mean differences (MD) along with 95% confidence intervals (CIs). Study heterogeneity was evaluated through the Q test and I^2^ statistic, considering values above 50% or *P*-values below 0.05 as indicative of significant heterogeneity. In such cases, a random-effects model was employed, with the between-study variance (τ^2^) estimated via the restricted maximum likelihood (REML) approach. Sensitivity analyses incorporated the Hartung-Knapp adjustment to yield more reliable confidence intervals. If substantial heterogeneity was detected (I^2^ > 50%), exploratory analyses were performed to investigate potential sources of variability, including subgroup analyses according to exercise modality and intervention duration, as well as meta-regression analyses based on participant characteristics.

The direction of effects was interpreted such that negative values indicated improvement in pain and disability scales (VAS, HAQ, TUG), whereas positive values reflected improvement in physical capacity (6MWT). To confirm the robustness of the findings, fixed-effect and random-effects models were compared, and sensitivity analyses were conducted excluding studies at high risk of bias. Statistical significance was set at *p* = 0.05. This interpretation was applied consistently across all tables and figures.

Publication bias was assessed using Egger’s regression asymmetry test and visual inspection of funnel plots. A *p*-value < 0.10 was considered suggestive of potential small-study effects, as this threshold provides greater sensitivity to detect bias in meta-analyses with a limited number of studies. All analyses were performed using Review Manager 5.4 (The Cochrane Collaboration, 2020) and STATA 17 (StataCorp LLC, Texas, USA).

## Results

### Study characteristics and descriptions

Thirteen studies met the eligibility criteria and were included in this systematic review, of which ten provided sufficient data for meta-analysis. The included studies, published between 2005 and 2024, involved a total of 548 participants (*n* = 277 intervention; *n* = 271 control). Nine studies were randomized controlled trials, and four were prospective controlled quasi-experimental studies.

Intervention types were distributed as follows: aquatic exercise (four studies), progressive resistance training (three studies), Tai Chi (two studies), and modified high-intensity interval training (HIIT) (four studies), with some studies combining modalities. Program duration ranged from 6 to 24 weeks, with a mean frequency of 2–3 sessions per week. All interventions were delivered in person by qualified healthcare professionals (physiotherapists or rheumatologists). The main characteristics of the studies included in the meta-analysis are summarized in Table 1.

**Table 1:** Main characteristics of the included studies and their outcomes

Study	Frequency	Duration per session	Intensity	Type of exercise/details	Supervision	Pain measure (VAS)	Function measure
Lyngberg et al. [15]	1 time/week	60 min	Low-moderate	Aerobic and muscle strength exercises. Warm-up, main exercise, stretching	Supervised by physiotherapist	VAS	HAQ
Nordgren et al. [16]	≥ 150 min/week (non-structured)	Variable (self-reported)	Light to moderate	Daily physical activity: walking, light resistance, daily activities	Not supervised	WOMAC	HAQ
Siqueira et al. [17]	3 times/week	45–60 min	Moderate (in water)	Aquatic exercise: warm-up, aerobics, strength, and stretching in pool	Supervised	VAS	6MWT
van den Ende et al. [18]	3 times/week	60–75 min	Moderate to intense	Combination of aerobic (bicycle, walking) and progressive resistance. Careful intensity control to avoid flare-ups	Supervised	VAS	HAQ, 6MWT
Flint Wagner et al. (2009)	2–3 times/week	60 min	Progressive, from low to moderate	Strength and resistance training with weights and elastic bands	Supervised	WOMAC	TUG
Strasser et al. [20]	3 times/week	Not specified	Moderate	Combined training: strength (isometric and dynamic) and aerobic (walking, bicycle)	Supervised	VAS	HAQ, 6MWT
Lourenzi et al. [21]	3 times/week	45–60 min	Progressive, increasing weekly load	Strength training with weights and machines, focus on lower limbs and core	Supervised	WOMAC	TUG, 6MWT
Orlova et al. [22]	≥ 3 times/week	Variable	Moderate	Prolonged program with aerobics, strength, and flexibility. In-person sessions and home exercises with personalized follow-up	Supervised and remote	VAS	HAQ, TUG
Bilberg et al. [23]	3 times/week	~ 30 min	High intensity (HIIT)	High-intensity intervals (30–60 s) alternated with active/passive recovery	Supervised	WOMAC	6MWT
Rall et al. [24]	3 times/week	60 min	Progressive, moderate	Strength training with weights and machines, focused on large muscle groups	Supervised	VAS	HAQ
Hu et al. [25]	≥ 3 times/week	Not specified	Variable, progressive	General recommendation for aerobic and strength exercise, with progression based on tolerance and response	Not applicable (review)	VAS,WOMAC	HAQ,6MWT,TUG
Uhlig et al. [26]	2–3 times/week	60 min	Soft to moderate	Tai Chi: postural control, breathing, balance, and relaxation	Supervised in group	VAS	6MWT,TUG
Lourenzi et al. [27]	3 times/week	45–60 min	Progressive, controlled	Progressive strength training with emphasis on plantar strength, use of dynamometry for adjustment	Supervised	WOMAC	HAQ,6MWT

*VAS*, Visual Analog Scale; *HAQ*, Health Assessment Questionnaire; *6MWT*, 6-min walk test; *TUG*, Timed Up and Go Test

### Meta-analysis results

The pooled results demonstrated that supervised exercise programs produced statistically significant and clinically relevant improvements in foot pain and physical function in RA patients compared to control groups. Functional outcomes were assessed using the HAQ, 6-min walk test, and Timed Up-and-Go, which primarily reflect global or lower-limb functional performance rather than strictly foot-specific function.

**Fig. 4:**
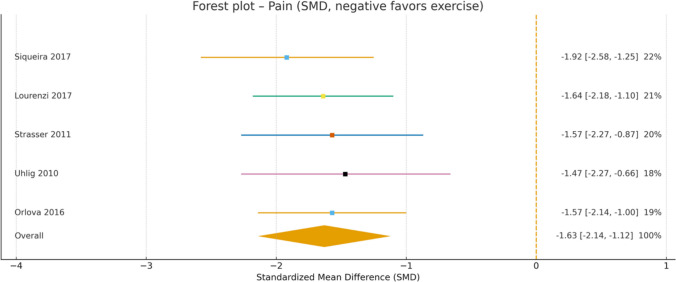
Forest plot showing standardized mean differences (SMD) for the effect of exercise on foot pain in patients with RA

**Fig. 5:**
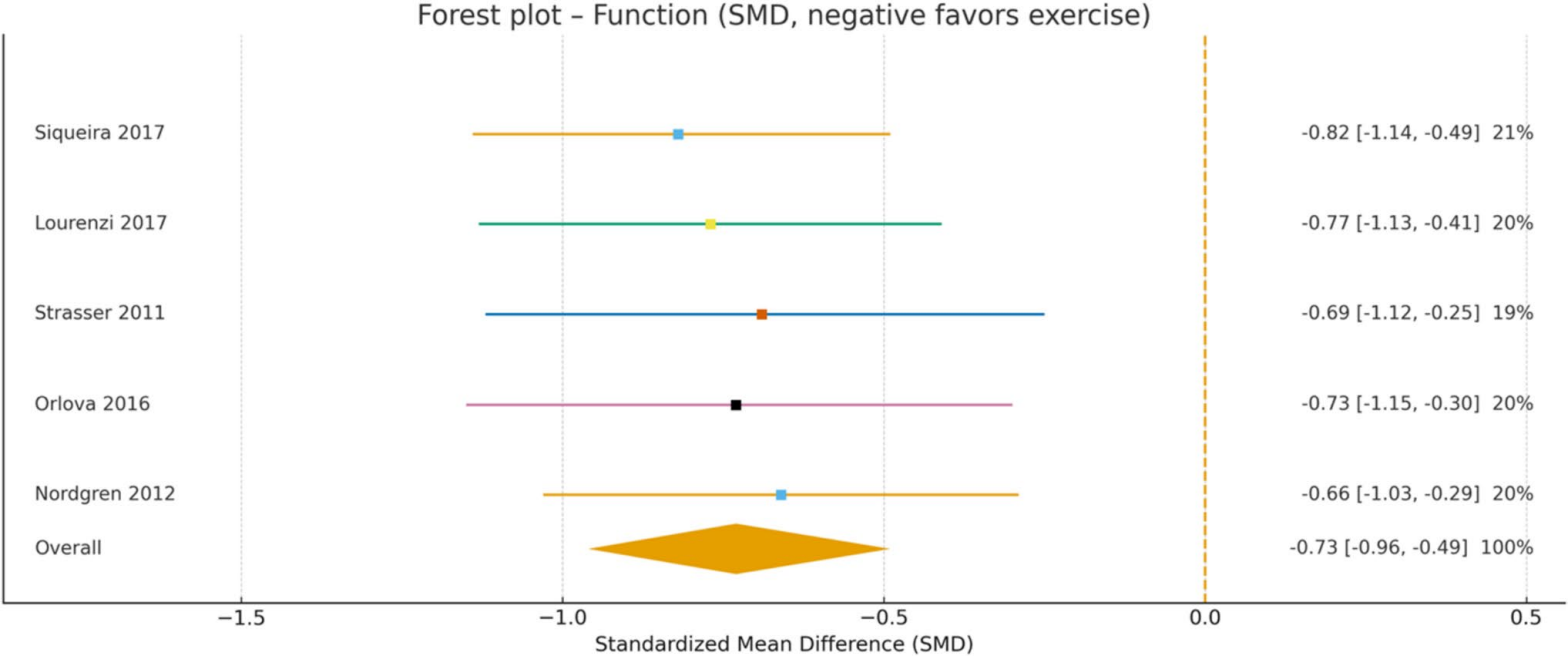
Forest plot showing standardized mean differences (SMD) for the effect of exercise on physical function in patients with RA

The forest plot for foot pain (Fig. 4) reveals substantial and consistent reductions across all included studies, with particularly strong effects observed for aquatic exercise [17] (SMD =  − 1.92) and progressive strength training [27] (SMD =  − 1.64). Similarly, the forest plot for physical function measured by HAQ (Fig. 5) demonstrates significant improvements across all studies, with a moderate and clinically meaningful overall effect (SMD =  − 0.73; 95% CI − 0.96 to − 0.49). Both medium and long-term interventions showed comparable benefits in functional capacity.

A complete summary of all analyzed outcomes, including the 6-min walk test and the Timed Up-and-Go test, is provided in Table 2. The certainty of the evidence (GRADE) was rated as moderate for all outcomes. The GRADE certainty of evidence was rated as moderate primarily due to methodological limitations across studies, including risks of bias related to blinding and outcome assessment, as well as heterogeneity in intervention protocols and outcome measures. No downgrading was applied for imprecision or publication bias, as effect estimates were consistent and confidence intervals did not cross the line of no effect.

**Table 2:** Effects of exercise on foot pain and function in adults with rheumatoid arthritis

Outcome	No. of studies	Pooled effect (95% CI)	*p*-value	Heterogeneity (I^2^)	Certainty (GRADE)
Foot Pain (VAS/WOMAC)	12	SMD = − 0.68 (− 0.89 to − 0.46)	< 0.001	49.7%	Moderate ⭑⭑⭑⭒
Function (HAQ)	5	SMD = − 0.73 (− 0.96 to − 0.49)	< 0.001	Low	Moderate ⭑⭑⭑⭒
6-Minute Walk Test (6MWT)	4	MD = + 47.6 m (31.4 to 63.8)	< 0.001	64%	Moderate ⭑⭑⭑⭒
Timed Up-and-Go (TUG)	4	SMD = −0.40 (− 0.59 to − 0.21)	< 0.001	Low to moderate	Moderate ⭑⭑⭑⭒

*CI*, confidence interval; *MD*, mean difference; *SMD*, standardized mean difference; *GRADE*, Grading of Recommendations Assessment, Development and Evaluation

### Heterogeneity assessment

Statistical heterogeneity was low to moderate for the analyses of foot pain (I^2^ = 49.7%), function-HAQ (I^2^ = low), and the TUG (I^2^ = low to moderate). Substantial heterogeneity was observed for the 6-min walk test (I^2^ = 64%), which is likely explained by clinical differences in exercise protocols (intensity, modality) and baseline patient characteristics. To further investigate sources of heterogeneity, meta-regression analyses were performed, which did not identify significant associations between the effect sizes and participant age, sex, or overall study quality.

### Sensitivity analysis

A comprehensive sensitivity analysis was conducted to assess the robustness of the meta-analysis findings. The initial analysis, which involved the sequential exclusion of studies with a high risk of bias [15, 19, 24], confirmed that the overall effect sizes for pain reduction and functional improvement remained stable and statistically significant. This indicates that the conclusions are not disproportionately influenced by lower-quality evidence.

Furthermore, the pooled estimates proved consistent when comparing fixed-effect and random-effects models, reinforcing the reliability of the results. To explore potential effect modifiers, analyses were stratified by methodological quality and intervention duration. The beneficial effect on functionality was nearly identical between studies of high methodological quality [16, 17, 22] (SMD =  − 0.74; 95% CI − 1.01 to − 0.47) and those of medium quality [20, 27] (SMD =  − 0.73; 95% CI − 1.12 to − 0.34). Similarly, the effect size was consistent for interventions lasting ≥ 12 weeks (SMD =  − 0.74) and those of shorter duration, under 12 weeks (SMD =  − 0.73).

An identical pattern was observed for the outcome of pain. The reduction in pain was robust, with comparable effect sizes in both high-quality studies (SMD =  − 0.65; 95% CI − 0.89 to − 0.41) and those of medium quality (SMD =  − 0.60; 95% CI − 0.95 to − 0.25). The duration of the intervention also showed a consistent effect, with sustained benefits in longer programs (SMD =  − 0.65) and clinically relevant improvements in shorter ones (SMD =  − 0.60).

### Risk of bias

The detailed results of the risk of bias assessment for each individual study are presented in Fig. 2. Overall, more than half of the included studies (54%) were judged to have a low risk of bias. A significant proportion (23%) raised some concerns, primarily due to issues in blinding procedures, while another 23% were classified as high risk, a category predominantly associated with older studies presenting several methodological limitations.

### Publication bias

**Fig. 6:**
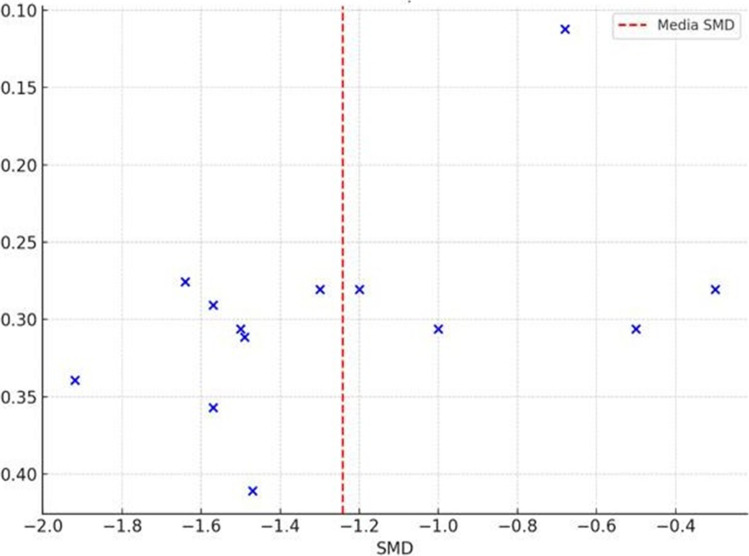
Funnel plot assessing publication bias for studies included in the meta-analysis of foot pain (VAS) in patients with RA

The funnel plot (Fig. 6) displays a reasonably symmetrical distribution of studies around the pooled effect size, suggesting no clear visual evidence of small-study effects.

To formally evaluate publication bias, Egger’s regression asymmetry test was performed, resulting in 0.91, a slope of − 0.43, and a *p*-value of 0.054. Although this *p*-value was slightly above the conventional threshold for statistical significance (*p* < 0.05), it does not provide strong evidence for publication bias. However, due to its proximity to the cutoff and the small number of studies included, the possibility of small-study effects cannot be fully excluded. Therefore, findings should be interpreted with a degree of caution.

## Discussion

This systematic review and meta-analysis demonstrates that structured therapeutic exercise is a safe and effective intervention for alleviating foot pain and improving physical function relevant to foot involvement in patients with RA. The pooled results, which showed clinically meaningful reductions in foot pain and improvements across functional measures like the HAQ, 6MWT, and TUG, are especially relevant given the high prevalence of foot involvement in RA and its critical role in maintaining autonomy and gait stability. Importantly, these benefits were achieved without exacerbating inflammatory activity.

Our findings provide a nuanced understanding of how different exercise modalities impact outcomes relevant to foot health. Among the evaluated options, aquatic exercise and HIIT demonstrated the strongest analgesic effects. This is likely because these modalities reduce joint loading while promoting movement in a low-impact environment, which may be particularly beneficial for pain management in patients with active foot involvement [25, 27]. In contrast, progressive resistance training and combined aerobic–strength programs yielded superior improvements in functional outcomes, such as gait performance, muscle strength, and joint stability. Tai Chi, although less effective for pure strength gains, provided significant benefits for balance, pain perception, and psychosocial well-being through its mind–body integration, particularly for older adults [28–30]. These findings align with previous evidence indicating that well-structured, protocolized exercise contributes not only to symptom control but also to broader health outcomes, including cardiorespiratory fitness and psychological well-being [31]. These findings extend previous general reviews on exercise in RA [11, 25] by specifically quantifying the benefits for foot health and comparing the efficacy of different modalities in this context. However, the heterogeneity of RA, including variability in disease stage, inflammatory activity, and comorbid conditions, underscores the necessity of individualized exercise prescriptions.

The differential effects observed across exercise modalities may be explained by the distinct mechanical and neuromuscular demands associated with each intervention. Aquatic exercise and Tai Chi are characterized by reduced joint loading, controlled movement patterns, and a strong emphasis on balance, breathing, and relaxation. These characteristics may facilitate pain reduction by minimizing mechanical stress on inflamed foot joints while promoting movement confidence and reducing pain-related fear, which is particularly relevant in patients with active foot involvement.

In contrast, resistance training and modified high-intensity interval training involve higher mechanical loads and greater neuromuscular activation of the lower limbs. These modalities may preferentially improve functional outcomes by enhancing muscle strength, power, and gait efficiency, thereby improving performance-based measures such as the HAQ, 6-min walk test, and Timed Up-and-Go. Although these interventions may be less immediately analgesic, the resulting gains in strength and physical capacity are likely to translate into improved functional independence.

Taken together, these findings suggest that the observed modality-specific effects are consistent with the functional demands and loading characteristics of each exercise type, supporting the rationale for tailoring exercise prescription according to both symptom burden and functional goals in patients with rheumatoid arthritis and foot involvement.

This evidence directly informs clinical practice. Exercise prescription for RA should begin with a comprehensive assessment, including disease classification (ACR/EULAR criteria), disease activity (DAS28), functional status (HAQ, TUG, 6MWT), comorbidity screening, and psychosocial factors influencing adherence [25, 31]. Based on this assessment, exercise can be tailored to disease activity: during remission or low activity (DAS28 < 3.2), moderate-to-vigorous resistance and aerobic exercise 2–3 times weekly is appropriate; for moderate activity (DAS28 3.2–5.1), low-impact modalities like aquatic therapy or Tai Chi should be prioritized; and during high disease activity or flares (DAS28 > 5.1), the focus should shift to gentle mobilizations and relaxation strategies to minimize joint overload [28–30]. Comorbidities further refine these choices; for instance, supervised HIIT can be considered for cardiovascular health, aquatic therapy is ideal for obesity and severe pain, resistance and balance training are key for osteoporosis and sarcopenia, and mind–body practices like Tai Chi benefit those with mood disorders or fatigue [25–30]. Exercise progression should follow a graded approach, guided by perceived exertion (Borg RPE 12–14) and symptom monitoring, with professional supervision enhancing safety, technique, and adherence [29, 30]. Regular reassessment every 4–8 weeks facilitates the adjustment of exercise parameters, and the integration of wearable technologies and telemedicine may further optimize long-term monitoring and patient engagement [25, 31].

From a clinical perspective, the findings of this review support a more targeted approach to exercise prescription in patients with rheumatoid arthritis and foot involvement. In individuals with predominant foot pain or moderate disease activity, low-impact modalities such as aquatic exercise or Tai Chi may be prioritized to reduce symptoms while minimizing joint loading. Conversely, in patients with low disease activity or stable disease, progressive resistance training or modified high-intensity interval training may be considered to enhance muscle strength, gait performance, and overall functional capacity.

Program duration and supervision also appear to be relevant for clinical implementation. Interventions lasting at least 12 weeks and delivered under professional supervision were associated with more consistent benefits, suggesting that structured, supervised programs may be preferable to unsupervised or short-term interventions, particularly in patients with established foot involvement.

Taken together, these findings highlight the importance of aligning exercise modality, intensity, and duration with individual patient characteristics, symptom burden, and functional goals, reinforcing the role of exercise as a core component of multidisciplinary care in rheumatoid arthritis.

Regarding limitations, an important consideration is that, while pain outcomes predominantly reflected foot-related symptoms, most functional outcomes included in this review were not strictly foot-specific. Measures such as the HAQ, 6-min walk test, and Timed Up-and-Go primarily assess global or lower-limb functional performance rather than isolated foot function. Although these outcomes are clinically relevant and strongly influenced by foot pain, deformity, and stability in patients with RA, the functional findings of this review should be interpreted as global or lower-limb functional proxies rather than direct measures of foot-specific function.

In addition, we identified substantial methodological heterogeneity across the included interventions, including variability in exercise modality, frequency, and intensity. The use of different outcome measures also limited direct comparability across studies. Although most studies were randomized, issues related to blinding, adherence, and incomplete reporting may have affected internal validity. Furthermore, relatively few studies included follow-up periods beyond 6 months, limiting conclusions regarding the long-term sustainability of the observed benefits.

Finally, despite the high clinical relevance of foot involvement in rheumatoid arthritis, few trials were specifically designed with foot-related outcomes as a primary focus. None of the included studies directly assessed objective inflammatory biomarkers, and conclusions regarding the safety of exercise interventions and the absence of disease exacerbation are therefore primarily based on the lack of reported clinical flares or adverse events rather than on biochemical evidence. Accordingly, interpretations related to inflammation and safety should be considered with caution. While no clear evidence of publication bias was observed, its potential influence cannot be entirely excluded.

Future research should therefore prioritize the adoption of standardized exercise protocols with explicit reporting of intensity, duration, and progression to allow for better comparison and replication. There is a need to carry out studies that prioritize on foot-specific outcomes, long-term follow-up, and the integration of patient-centered measures, including quality of life and adherence, as well as develop validated clinical algorithms to guide individualized exercise prescription based on disease.

## Conclusion

This systematic review and meta-analysis suggests that supervised exercise interventions are associated with reductions in foot-related pain and improvements in physical function relevant to foot involvement in patients with rheumatoid arthritis. Aquatic exercise and Tai Chi appear to be particularly beneficial for pain reduction, while resistance training and modified high-intensity interval training are more consistently associated with improvements in functional performance. However, these findings should be interpreted within the context of the available evidence, as most functional outcomes reflect global or lower-limb performance rather than strictly foot-specific function.

Taken together, the results support the integration of structured exercise into multidisciplinary care for rheumatoid arthritis, with exercise modality selection guided by symptom burden and functional goals, rather than implying modality-specific effects on isolated foot function.

## Data Availability

The data are available upon request from the author.
